# LATINX GRANDPARENTS RAISING GRANDCHILDREN: THE INFLUENCE OF CULTURAL NORMS

**DOI:** 10.1093/geroni/igac059.1031

**Published:** 2022-12-20

**Authors:** Nancy Mendoza, Cherrie Park

**Affiliations:** The Ohio State University, Columbus, Ohio, United States; The Ohio State University, Columbus, Ohio, United States

## Abstract

Few studies have focused on the experiences of Latinx grandparents. Using a grounded theory approach, nine Latinx grandparent caregivers were interviewed in an effort to understand how the Latinx culture influences their experiences. Major themes that emerged during data analysis were related to reasons for caring. These included cultural norms of caring and reasons for the non-usage of services. Findings suggest Latinx grandparents tend to care for their grandchildren because they view it as part of their role as a grandparent. Thus, the biggest “barrier” to acquiring services could be that they do not view themselves as primary caregivers. These findings emphasize the need to understand the experiences of these grandparents and the importance of tailoring education and resources to this subgroup. This study provides professionals with a new way of looking at services for Latinx grandparents and opens the door to a new set of implications for practice.

